# A Divergent Sequela of Scleral Buckle Removal

**DOI:** 10.7759/cureus.60227

**Published:** 2024-05-13

**Authors:** Leroy Tan, Fiona Chew Lee Min, Zabri B Kamarudin, Safinaz Mohd Khialdin

**Affiliations:** 1 Ophthalmology, Universiti Kebangsaan Malaysia Medical Centre, Kuala Lumpur, MYS; 2 Ophthalmology, Sunway Medical Centre Velocity, Kuala Lumpur, MYS; 3 Ophthalmology, Hospital Selayang, Batu Caves, MYS; 4 Ophthalmology, Hospital Universiti Kebangsaan Malaysia, Kuala Lumpur, MYS

**Keywords:** vitreo-retinal surgery, squint surgery, scleral buckle migration, strabismus surgery, scleral buckle removal, scleral buckle, scleral buckling

## Abstract

Scleral buckle (SB) removal is done for a variety of reasons following treatment of rhegmatogenous retinal detachments (RRD), such as buckle exposure, migration, and infection. The most worrying complication of SB removal is retinal redetachment. We report a unique case of a patient developing strabismus about one month after scleral buckle removal for anterior migration and exposure of the scleral buckle. We also share a successful strabismus surgery which had a main aim of relieving diplopia in the patient's primary gaze.

## Introduction

Scleral buckle (SB) removal is done for a variety of reasons following treatment of rhegmatogenous retinal detachments (RRD), such as buckle exposure, migration, and infection [1,2]. Strabismus itself can be caused by an in-situ scleral buckle [1]. The most worrying complication of SB removal is retinal redetachment [1]. This case report describes an uncommon development of strabismus in an adult patient as a complication of SB removal.

This case was previously presented as a poster at the 7th USM Ophthalmology Symposium in conjunction with the 9th Conjoint Ophthalmology Scientific Conference, on 13th September 2019.

## Case presentation

A 50-year-old gentleman, with no medical illnesses had an encircling SB removal done for RRD in the right eye (RE) 5 years prior and postoperatively enjoyed good vision with the treated eye. He had presented acutely with a complaint of red eye, eye discharge, and pain to the vitreoretinal clinic. He reported no diplopia. At that time, the SB was found to be anteriorly migrated throughout and there was an exposed area at the supero-temporal aspect of the RE. He was admitted for intravenous antibiotics and SB removal was done.

**Figure 1:**
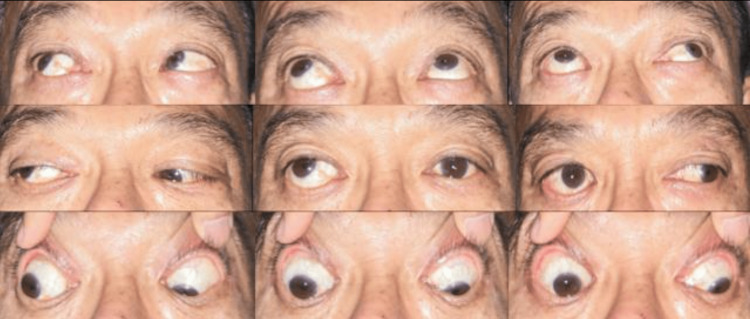
Alternating exotropia with limited depression and adduction of the right eye one month after scleral buckle removal surgery for buckle anterior migration and exposure

At the one-month follow-up visit after SB removal, he complained of a progressively worsening double vision. On examination, visual acuity was 6/24 and 6/9 in his right and left eye, respectively, with no measurable stereopsis. There was no pain on eye movement. He had an alternating exotropia of 40-prism diopters (PD) and hypertropia of 30-PD. There was also limited depression and adduction of the RE. There were no signs of re-detachment. Figure 1 below shows the 9-gaze view at the one-month follow-up visit after SB removal.

Magnetic resonance imaging of the orbits revealed intact extraocular muscles (EOM). His thyroid function tests were normal. Eight months after the initial SB removal, the patient underwent bilateral lateral rectus recession of 7.5mm and RE superior rectus recession of 7.0mm. Intraoperatively, a forced duction test revealed limited adduction and depression. The lateral and superior recti were adherent to the globe with massive fibrosis.

**Figure 2:**
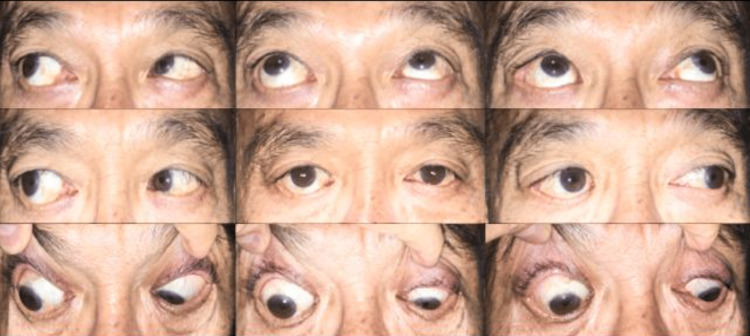
The patient was orthophoric in primary position at the one-year follow-up visit after squint surgery

One week after squint surgery, the patient's diplopia resolved, and at the one-year follow-up, he was orthophoric with no EOM restriction and no signs of retinal redetachment. This patient is to be seen at least yearly to monitor for re-detachment. Figure 2 below shows the 9-gaze view at the one-year follow-up visit post-squint surgery.

## Discussion

Strabismus development after buckle surgery for retinal detachment repair is fairly common [2]. Removal of SB is sometimes done to improve strabismus following buckle surgery. The mechanisms explaining this as discussed by Chang et al. are direct recti muscle attachment to the sclera, lengthening of muscles over bulky implants, and freeing of a muscle inadvertently incorporated in the scleral buckle. Interestingly, the lengthening of muscles over bulky implants can contribute to the development of squint following buckle removal. This can usually be improved upon removal of the buckle [3]. Unique to this case is that our patient developed a squint and diplopia one month after the scleral buckle was removed. To the best of our knowledge, no reports of strabismus developing post-scleral buckle removal such as this have been documented. 

We postulate two possible mechanisms that could explain the development of strabismus in this case. Firstly, the infection itself caused localized fibrosis at the site of infection which in turn led to restricted function of the lateral and superior recti [1,4]. Secondly, the mechanical stretching of the inferior and medial recti due to anterior migration of the scleral buckle had caused permanent lengthening of the recti muscles. Upon removal of the scleral buckle, the ability of the recti muscles to initiate movement of the globe is thus reduced in proportion to the amount of lengthening [1,2].

The amount of recession performed was decided based on standardized tables as described by Wright [5]. Anatomical changes from scleral buckle surgery may lead to the inaccuracy of using standard tables for strabismus surgery [6]. In our case, however, we found that using Wright’s prescribed values had afforded us a fairly good outcome. More such cases need to be reported before we can come to a definitive answer to this.

## Conclusions

The development of strabismus as a complication following scleral buckle removal is unique. This was likely the result of fibrosis secondary to infection, or mechanical stretching of the EOMs as a result of anterior migration of the SB. Treatment with strabismus surgery can successfully correct diplopia in cases such as these.
